# Supplementary material for: MetaRibo-Seq measures translation in microbiomes
Source: Nat Commun. 2020 Jun 29;11:3268. doi: 10.1038/s41467-020-17081-z (PMC7324362; doi:10.1038/s41467-020-17081-z)
Supplement: Supplementary file 10 — Supplementary Data 7 [file 41467_2020_17081_MOESM10_ESM.zip › File2/Confidence_High_Taxonomy/103391_out.krona.html]

Javascript must be enabled to view this page.

members
magnitude
magnitudeUnassigned
count
unassigned
taxon
rank

103391\_out

3

superkingdom
3
2

3
phylum
1239

186801
class
3

order
3
186802

1
family
186806

1730
1
genus

species
1

SRS144506\_contig\_number\_contig-100\_7331.320053
39496

2
family
186803

841
1
genus


SRS043411\_contig\_number\_contig-100\_4432.113344
360807
species
1

genus
1
28050


SRS013951\_contig\_number\_contig-100\_22589.120851
28052
species
1
